# A screening of wild bird samples enhances our knowledge about the biodiversity of avian adenoviruses

**DOI:** 10.1007/s11259-022-09931-6

**Published:** 2022-06-06

**Authors:** Balázs Harrach, Annamária Megyeri, Tibor Papp, Krisztina Ursu, Sándor A. Boldogh, Győző L. Kaján

**Affiliations:** 1 Veterinary Medical Research Institute, Eötvös Loránd Research Network, Budapest, Hungary; 2 Veterinary Diagnostic Directorate, National Food Chain Safety Office, Budapest, Hungary; 3 Directorate, Aggtelek National Park, Jósvafő, Hungary

**Keywords:** Adenovirus, Molecular typing, Screening, Wild birds

## Abstract

Wild birds are threatened by anthropic effects on a global scale, and their adenoviruses might contribute to their endangerment. Thus, it is important to reveal the real biodiversity of avian adenoviruses, as, unfortunately, this research topic is far from being prioritized. The turkey hemorrhagic enteritis is an economically important disease causing high mortalities, and its causative siadenoviral agent is only distantly related to other avian siadenoviruses in phylogenetic analyses. Both to enhance our knowledge about the biodiversity of wild bird adenoviruses and to possibly trace back the origin of the turkey hemorrhagic enteritis virus, numerous Hungarian wild bird samples were screened for adenoviruses using PCR, and the detected strains were typed molecularly. The screening revealed numerous new adenovirus types, several of which represent novel adenovirus species as well, in the genera *Atadenovirus*, *Aviadenovirus* and *Siadenovirus*.

## Introduction

Adenoviruses are double-stranded DNA viruses with an icosahedral capsid, where the major capsid proteins are the hexon, the penton base and the protruding fiber (Gallardo et al. 2021). The family *Adenoviridae* consists of six genera: genus *Aviadenovirus, Atadenovirus, Ichtadenovirus, Mastadenovirus, Siadenovirus* and *Testadenovirus* (Benkő et al. 2022). The members of three from these six genera may infect birds (Harrach et al. 2019). Historically, all avian adenoviruses were, and numerous strains still are classified into the genus *Aviadenovirus*. But based on phylogenetic, genome structural and evolutionarily research, the genera *Atadenovirus* and *Siadenovirus* have been established for the divergent types. E.g., the egg drop syndrome virus (duck adenovirus 1) is a well-known and economically important member of the genus *Atadenovirus* (Harrach et al. 1997).

Adenoviruses of wild birds are less known and characterized compared to those of domesticated birds. However, these viruses might threaten not only their own host species, which are often endangered already due to habitat loss or other negative anthropic effects but farm and companion animals as well. Although most adenoviruses infect a single or a narrow range of host species, host switches do occur and result usually in elevated pathogenicity (Kaján et al. 2020).

The turkey hemorrhagic enteritis is an economically important disease causing as high as 60% mortalities in unvaccinated flocks. Its causative siadenoviral agent (turkey adenovirus 3) is only distantly related to other avian siadenoviruses in phylogenetic analyses, and all other identified turkey adenovirus types (1, 2, 4 and 5) are aviadenoviruses (Kaján et al. 2010; Marek et al. 2014). Based on the elevated level of pathogenicity and a yet untraceable evolutionary origin of the virus, a relatively recent host switch can be hypothesized, but the original host species is unknown.

Both to enhance our knowledge about the biodiversity of wild bird adenoviruses and to possibly trace back the origin of the turkey hemorrhagic enteritis virus, we screened numerous wild bird samples for adenoviruses, and the detected strains were typed molecularly.

## Materials and methods

### Wild bird samples

Samples (n = 82) originated mainly from two Hungarian sources. Wild bird carcasses (n = 46) were collected in the Aggtelek National Park by park directorate staff between 2010 and 2014. Cloacal swabs (n = 33) were also collected in the same national park in the wild bird ringing station of the Bódva Valley, Szalonna, Hungary organized and supervised also by park directorate staff on 24 Aug 2020. Due to the size of the country and the high mobility of the birds, samples of this region represent the majority of the Hungarian avian fauna. Further three parrot carcasses originated from a pet shop. Sampled species are summarized in Table 1. Samples were stored frozen at -20 °C until Sept 2021, when carcasses were dissected and liver, kidney and gut samples were homogenized in 1 ml phosphate-buffered saline, and swab samples were also soaked in 1 ml phosphate buffered saline for 1 h. Both sample types were centrifuged for 5 min on 13.000 ×*g*.

**Table 1:** Screened wild bird samples

Species	Number of swab samples	Number of organ samples
barn swallow (*Hirundo rustica*)	0	1
black redstart (*Phoenicurus ochruros*)	1	0
Bohemian waxwing (*Bombycilla garrulus*)	0	7
common blackbird (*Turdus merula*)	1	1
common chaffinch (*Fringilla coelebs*)	3	0
common kestrel (*Falco tinnunculus*)	0	1
common kingfisher (*Alcedo atthis*)	1	0
common linnet (*Linaria cannabina*)	0	1
common nightingale (*Luscinia megarhynchos*)	1	0
common pigeon (*Columba livia*)	0	1
common starling (*Sturnus vulgaris*)	0	1
Eurasian blackcap (*Sylvia atricapilla*)	9	0
Eurasian blue tit (*Cyanistes caeruleus*)	9	0
Eurasian coot (*Fulica atra*)	0	1
Eurasian jay (*Garrulus glandarius*)	0	1
Eurasian nuthatch (*Sitta europaea*)	0	1
Eurasian sparrowhawk (*Accipiter nisus*)	0	4
Eurasian tree sparrow (*Passer montanus*)	0	3
Eurasian treecreeper (*Certhia familiaris*)	1	0
European bee-eater (*Merops apiaster*)	0	1
European goldfinch (*Carduelis carduelis*)	0	1
European green woodpecker (*Picus viridis*)	1	0
European robin (*Erithacus rubecula*)	2	0
European serin (*Serinus serinus*)	0	1
fieldfare (*Turdus pilaris*)	0	1
great spotted woodpecker (*Dendrocopos major*)	0	1
great tit (*Parus major*)	2	1
green-cheeked parakeet (*Pyrrhura molinae*)	0	2
hawfinch (*Coccothraustes coccothraustes*)	0	3
hooded crow (*Corvus cornix*)	0	1
lesser whitethroat (*Sylvia curruca*)	1	0
little owl (*Athene noctua*)	0	2
long-eared owl (*Asio otus*)	0	2
nanday parakeet (*Aratinga nenday*)	0	1
red-backed shrike (*Lanius collurio*)	0	2
song thrush (*Turdus philomelos*)	0	3
strigiformes species	0	1
willow tit (*Poecile montanus*)	0	1
willow warber (*Phylloscopus trochilus*)	1	0
yellowhammer (*Emberiza citrinella*)	0	2
**SUM**	**33**	**49**

### PCR and sequencing

DNA was extracted from homogenate or swab supernatants using the Bioextract Superball (Biosellal) kit. The samples were screened for adenoviruses using a nested PCR detecting all adenoviruses (Kaján 2016; Wellehan et al. 2004). 321-bp-long PCR products of the second round were Sanger sequenced on both strands. Sequences were deposited in the NCBI Nucleotide database under accession numbers OL603899-OL603913.

### Phylogenetic analysis

Sequence reads were assembled and translated to 90-amino acids-long protein sequences in Geneious. Only a single one was included in the phylogenetic analyses if multiple strains were identified with identical DNA polymerase amino acid sequences. The nucleic acid sequence identity of the available partial DNA polymerase gene was calculated for these strains. To aid proper phylogenetic placement, the complete DNA polymerase amino acid sequences were used from the reference strains for the tree inference where available (e.g., the DNA polymerases originating from complete genomes), and these were aligned with the partial coding sequences. Amino acid sequences were aligned using the MAFFT G-INS-i algorithm. The alignment was edited manually: the edited alignment length was 1024 amino acids (because of the used complete DNA polymerase sequences). Missing parts of the partial DNA polymerase sequences were substituted using gaps. The LG + I + G evolutionary model was applied selected using ModelTest-NG v0.1.5. The best phylogenetic tree was chosen from 300 replicates inferred using RAxML-NG v1.0.1, and the robustness of the tree was determined with a non-parametric bootstrap calculation using 1000 repeats. The transfer bootstrap expectation values were applied to the tree. The phylogenetic tree was visualized using MEGA 7, and it was rooted on the midpoint, and bootstrap values were given as percentages if they reached 75%.

To determine the amino acid sequence identity of the virus strains compared to different adenoviral species, a pairwise sequence identity analysis was also conducted based on the partial DNA polymerase sequences using the Sequence Demarcation Tool v1.2.

In both analyses, all at-, avi- and siadenovirus species were included, and all were represented by a single member. Furthermore, the virus strains most closely related to the newly detected viruses were also included in the analyses. These were pinpointed using the blastx service of NCBI on 27 Oct 2021.

## Results

### Positivity

From the screened 82 wild bird samples, adenoviruses were detected in 16 (20%). The positivity rate of organ samples was 27%, whereas that of the swab samples was 9%.

### Phylogenetic analysis

**Fig. 1:**
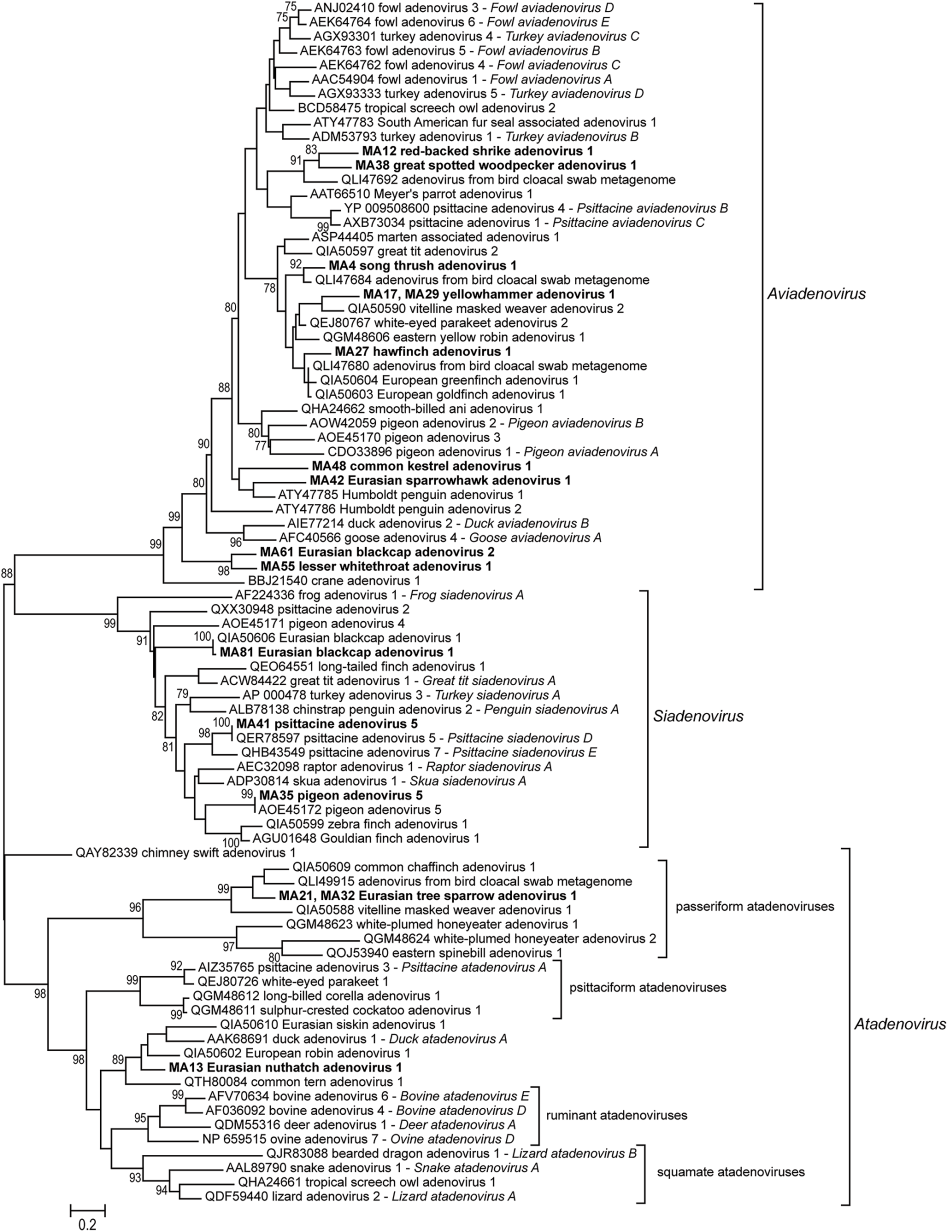
Phylogenetic analysis of wild bird adenoviruses. Newly detected adenovirus strains are in bold

The phylogenetic tree is presented in Fig. 1. From the detected adenoviruses, ten strains were classified as aviadenoviruses, and three as at-, three as siadenoviruses. Samples MA17 and MA29 (both yellowhammers) contained adenoviruses with 100% nucleic acid identity on the analyzed stretch. Between samples MA21 and MA32 (both Eurasian tree sparrows), one nucleotide difference was observed which is a silent mutation only.

Pairwise sequence identities to members of the closest related officially accepted adenoviral species are summarized in Table 2. Psittacine adenovirus 5 (species *Psittacine siadenovirus D*) was detected in a nanday parakeet (*Aratinga nenday*) with 100% sequence identity on the analyzed partial DNA polymerase amino acid sequence. For other detected strains, the sequence identities ranged between 55.6 and 77.8%.

**Table 2:** The highest pairwise amino acid sequence identity of the partial viral DNA polymerase sequences compared to all avi-, at- and siadenoviral species

	Analyzed strain’s			Closest related officially accepted adenovirus species member	Pairwise sequence identity
	**adenovirus type**	**host species**	**strain identifier**		
**genus** ***Aviadenovirus***					
	Eurasian sparrowhawk adenovirus 1	Eurasian sparrowhawk	MA42	*Fowl aviadenovirus B* (fowl adenovirus 5)	77.8%
	common kestrel adenovirus 1	common kestrel	MA48	*Fowl aviadenovirus A* (fowl adenovirus 1)	77.8%
	hawfinch adenovirus 1	hawfinch	MA27	*Fowl aviadenovirus A* (fowl adenovirus 1)	75.6%
	song thrush adenovirus 1	song thrush	MA4	*Fowl aviadenovirus E* (fowl adenovirus 6)	72.2%
	red-backed shrike adenovirus 1	red-backed shrike	MA12	*Fowl aviadenovirus B* (fowl adenovirus 5)	72.2%
	yellowhammer adenovirus 1	yellowhammer	MA17, MA29	*Fowl aviadenovirus D* (fowl adenovirus 3)	71.1%
	great spotted woodpecker adenovirus 1	great spotted woodpecker	MA38	*Turkey aviadenovirus B* (turkey adenovirus 1)	71.1%
	lesser whitethroat adenovirus 1	lesser whitethroat	MA55	*Duck aviadenovirus B* (duck adenovirus 2)	68.9%
	Eurasian blackcap adenovirus 2	Eurasian blackcap	MA61	*Fowl aviadenovirus D* (fowl adenovirus 3)	68.9%
**genus** ***Atadenovirus***					
	Eurasian nuthatch adenovirus 1	Eurasian blackcap	MA13	*Duck atadenovirus A* (duck adenovirus 1)	77.8%
	Eurasian tree sparrow adenovirus 1	Eurasian tree sparrow	MA21, MA32	*Snake atadenovirus A* (snake adenovirus 1)	55.6%
**genus** ***Siadenovirus***					
	psittacine adenovirus 5	nanday conure	MA41	*Psittacine siadenovirus D* (psittacine adenovirus 5)	100.0%
	pigeon adenovirus 5	common pigeon	MA35	*Skua siadenovirus A* (South Polar skua adenovirus 1)	76.7%
	Eurasian blackcap adenovirus 1	Eurasian blackcap	MA81	*Raptor siadenovirus A* (raptor adenovirus 1)	71.9%

## Discussion

Mostly due to anthropic effects, the global avifauna has been suffering a constant decline in recent decades (Burns et al. 2021). And still, wild bird adenoviruses are far from being well known and characterized, though these might contribute to the endangerment of different avian species. A high level of positivity was observed in our samples, and from the 14 different adenovirus types detected, only three were known to science: the pigeon adenovirus 5, the psittacine adenovirus 5 and the Eurasian blackcap adenovirus 1 (Ballmann and Harrach 2016; Katoh et al. 2009; Rinder et al. 2020). But even from the latter host species, a novel type could be described also after the screening of mere nine samples.

Though progress is evident in the research of wild bird adenoviruses in recent years (Niczyporuk et al. 2020; Phalen et al. 2019; Rinder et al. 2020; Sarker 2021; Vaz et al. 2020), perhaps because of the high number of avian species and biased research towards human and domesticated animal viruses, the number of unknown wild bird adenoviruses seems to be still enormous. And because of these lacking puzzle pieces, it is hard to classify the found strains properly. From the 14 new strains found, only a single one could be classified into an established adenoviral species. All the other strains showed extensively lower sequence identity to the closest related adenoviral species than determined by the species demarcation criterion: 85–90% (Benkő et al. 2022); thus, all these strains will represent novel adenoviral species.

These low sequence identities were not only observed in this analysis in comparison to the accepted species. Similarly low identity values were recorded in a blastx analysis against the complete GenBank as well in most cases (data not shown). Based on this phenomenon, though the closest blastx hits were included in the phylogenetic analysis (Fig. 1), these hits did not help in classifying the samples or tracing their evolutionary origin and relatedness. Most found strains are placed on a relatively long branch in the tree reconstruction, meaning that evolutionarily close relatives could not be included in the analysis. The observation that the newly discovered avian adenoviruses are highly divergent and therefore it is likely that there is far more adenovirus diversity to be found, is not a novel concept and has recently been emphasized by the publications of Athukorala et al. (2020, 2021) and Vaz et al. (2020).

The only exception available, where coevolution can be hypothesized for any of the newly detected strains, is that of the finch aviadenoviruses. The only finch aviadenoviruses in the tree are the viruses of the hawfinch, the European greenfinch and the European goldfinch (Rinder et al. 2020). These strains are closely related and form a monophyletic clade. There is a fourth virus strain in the clade originating from an unknown avian species (NCBI Protein: QLI47680). As pairwise sequence identities within this clade were always above 85% (data not shown), the establishment of a common finch aviadenovirus species might be suggestable, also supported by the related host species.

Another phenomenon, most possibly caused by the current lack of data from closely related viral strains, is that of the chimney swift atadenovirus (Needle et al. 2019). Though monophyletic with atadenoviruses, it is placed on the most basal branch of this genus, far from the well-demarcated three clades of other avian atadenoviruses. From these three, coevolution-based monophyletic clades can be observed in at least two: in that of the passeriform and the psittaciform atadenoviruses. The chimney swift is evolutionarily not closely related to these avian clades, thus the distance of its adenovirus is acceptable to a certain degree. But such a basal placement is not justified by the sequence identity values. The lowest pairwise sequence identity of 44.0% was measured between the sulphur-crested cockatoo adenovirus 1 (QGM48611) and the common chaffinch adenovirus 1 (QIA50609) in the *Atadenovirus* genus (Rinder et al. 2020; Vaz et al. 2020), whereas the chimney swift atadenovirus 1 shared 64.4% sequence identity with the common tern adenovirus 1 (Kraberger et al. 2022). The used 90-amino acids-long stretch is adequate for a genus and a preliminary species level typing usually and employed excessively for this purpose (Kaján et al. 2011; Kaján 2016). Still, in this case, further genomic data is needed for the proper phylogenetic analysis and safe classification of this chimney swift strain.

The results supported the already described host species in two cases and broadened the host range in one. Pigeon adenovirus 5 was detected from a common pigeon (*Columba livia*) again. It had been detected only once previously (Ballmann and Harrach 2016), so the host species was confirmed for this virus. The same is true for the Eurasian sparrowhawk adenovirus 1, as it had been detected 13 further times from the same host species (*Accipiter nisus*) (unpublished). Psittacine adenovirus 5 has been detected already from Pacific parrotlet (*Forpus coelestis*), sun conure (*Aratinga solstitialis*), cockatiel (*Nymphicus hollandicus*) and budgerigar (*Melopsittacus undulatus*) (Cassmann et al. 2019; Gall et al. 2020; Gottdenker et al. 2019; Katoh et al. 2009) and now from a nanday conure (*Aratinga nenday*). This type, similarly to psittacine adenovirus 2, seemingly has a broad host range within the order Psittaciformes.

Though we were also aiming to trace back the possible origin of turkey adenovirus 3, unfortunately, closely related adenoviral strains could not be detected in the wild bird samples. Perhaps North American wild bird samples should be screened, as the host species originates from there, and the disease was detected in Minnesota for the first time.

Wild birds are threatened by anthropic effects on a global scale, and their diverse adenoviruses might contribute to their endangerment. The screening of Hungarian wild bird samples revealed numerous new adenovirus types, several of which may represent novel adenovirus species as well, in the genera *Atadenovirus*, *Aviadenovirus* and *Siadenovirus*.

## Data Availability

Sequences were deposited in the NCBI Nucleotide database under accession numbers OL603899-OL603913.
